# *Helicobacter pylori* infection: a dynamic process from diagnosis to treatment

**DOI:** 10.3389/fcimb.2023.1257817

**Published:** 2023-10-19

**Authors:** Qifang Sun, Chengzhi Yuan, Sainan Zhou, Jing Lu, Meiyan Zeng, Xiong Cai, Houpan Song

**Affiliations:** ^1^ School of Traditional Chinese Medicine, Hunan University of Chinese Medicine, Changsha, Hunan, China; ^2^ Hunan Provincial Key Laboratory of Traditional Chinese Medicine (TCM) Diagnostics, Hunan University of Chinese Medicine, Changsha, Hunan, China; ^3^ School of Medicine, Hunan University of Chinese Medicine, Changsha, Hunan, China; ^4^ The First Hospital of Hunan University of Chinese Medicine, Changsha, Hunan, China; ^5^ School of International Education, Hunan University of Chinese Medicine, Changsha, Hunan, China

**Keywords:** *Helicobacter pylori*, pathogenesis, diagnostic strategy, treatment, antibiotic resistance

## Abstract

*Helicobacter pylori*, a gram-negative microaerophilic pathogen, causes several upper gastrointestinal diseases, such as chronic gastritis, peptic ulcer disease, and gastric cancer. For the diseases listed above, *H. pylori* has different pathogenic mechanisms, including colonization and virulence factor expression. It is essential to make accurate diagnoses and provide patients with effective treatment to achieve positive clinical outcomes. Detection of *H. pylori* can be accomplished invasively and noninvasively, with both having advantages and limitations. To enhance therapeutic outcomes, novel therapeutic regimens, as well as adjunctive therapies with probiotics and traditional Chinese medicine, have been attempted along with traditional empiric treatments, such as triple and bismuth quadruple therapies. An *H. pylori* infection, however, is difficult to eradicate during treatment owing to bacterial resistance, and there is no commonly available preventive vaccine. The purpose of this review is to provide an overview of our understanding of *H. pylori* infections and to highlight current treatment and diagnostic options.

## 1 Introduction

*Helicobacter pylori*, a well-known bacterium that colonizes the human stomach, has gained increasing attention in the last 40 years. This bacterium is a flagellated, gram-negative bacillus with infection rates reaching 80–90% in some populations. There are large variations in *H. pylori* prevalence across the globe. Infections by *H. pylori* are ubiquitous in developing countries, reaching the highest rate of 80% or more among adults in Africa, followed by Latin America (63.4%) and Asia (54.7%) (Smith et al., 2018). According to the latest available estimates, approximately 589 million people in mainland China are infected with *H. pylori*, representing a prevalence of 44.2% (Ren et al., 2022). North America has one of the lowest prevalence rates of 37.1%. Infections by *H. pylori* are more prevalent in Hispanic and Native American individuals in the United States, followed by Afro-Americans and Caucasians (Hooi et al., 2017). However, in general, *H. pylori* prevalence is declining in highly industrialized countries of the Western world.

The spread of *H. pylori* can occur directly from one person to another person or indirectly from an infected person to the environment. Moreover, *H. pylori* DNA has been detected in human feces, saliva, and supragingival plaque, suggesting a fecal-oral and oral-oral route of transmission. High-pressure profession, water supplies, smoking, and dietary habits have been associated with a higher risk of *H. pylori* acquisition. It has also been suggested that gut microbiota may contribute to intrafamilial transmission of *H. pylori* (Leja et al., 2019). Many studies have demonstrated that developing countries have a high prevalence of *H. pylori* with a much higher recurrence rate than that of developed countries (Hu et al., 2017). It is possible that a therapeutic scheme with low eradication rates, insufficient treatment times, *H. pylori* oral colonization, *H. pylori* coccoid forms, and *H. pylori* biofilm will result in a recurrence of *H. pylori* (Sun and Zhang, 2019).

Various diseases are associated with *H. pylori* infection, especially gastrointestinal ones. Clarifying the pathogenic characteristics and mechanisms of infection of *H. pylori* is crucial for the effective treatment of *H. pylori*-related gastrointestinal diseases. Nevertheless, within the digestive system research community, there persist certain contentions pertaining to the pathogenesis, virulence factors, clinical classification, therapeutic approaches, and mechanisms of drug resistance associated with *H. pylori*. Consequently, it is imperative to comprehensively consolidate existing research in the aforementioned domains. In this paper, we reviewed the related disorders produced by *H. pylori* and explained the pathogenic mechanism centered on its colonization and virulence factor expression. Moreover, we described invasive and non-invasive diagnostic methods for *H. pylori* infection. The treatment options for eradicating *H. pylori* include traditional experiential therapies, such as bismuth quadruple therapy, and adjuvant therapies, such as probiotics and traditional Chinese medicine (TCM). We also introduced research on the drug resistance of *H. pylori*, thereby providing a deeper understanding on this bacterium. Finally, we discussed other *Helicobacter* species and their treatment and diagnosis.

## 2 Clinical diseases

### 2.1 Gastric diseases

It is well known that *H. pylori* infection is associated with gastrointestinal diseases, such as gastritis, peptic ulcer disease, gastric mucosa-associated lymphoid tissue (MALT) lymphoma, and gastric cancer. Gastritis, also known as chronic gastritis, is characterized by chronic inflammation of the gastric mucosa. An *H. pylori* infection is one of the most common causes of chronic gastritis. Eighty patients with dyspepsia and chronic gastritis were evaluated by Fatema et al. (2015). Among them, 67 (83.8%) were infected with *H. pylori*, whereas 13 (16.2%) were not. As a result of its features and enzymatic properties, *H. pylori* can survive in acidic stomach environments and has specific virulence factors that increase the risk of gastric disease by targeting different cellular proteins to regulate inflammatory responses in the host (Kim and Wang, 2021). Umit et al. (2009) found that high cytotoxin-associated antigen A (CagA) positivity in *H. pylori* strains is associated with more severe gastritis, according to some histological features of the disease. *H. pylori* genotypes also differ significantly among populations in different regions; for instance, a greater prevalence of VacA, CagA^+^, and BabA2^−^ genotypes is observed in patients with chronic gastritis in southern Mexico (Atrisco-Morales et al., 2018).

The development of gastric cancer occurs gradually from normal gastric mucosa, superficial gastritis, gastritis with atrophy, intestinal metaplasia, and/or atypical hyperplasia. Infection with *H. pylori* plays a significant role in gastric cancer. Previous research has established that its worldwide attributable fraction in non-cardia gastric cancer (NCGC) increased from 74.7% to 89.0%. This meant that approximately 120,000 additional NCGC cases were associated with *H. pylori* infection for a total of about 780,000 cases (Plummer et al., 2015). As a result of chronic gastritis and gastric ulcers caused by *H. pylori* infection, the DNA of gastric mucosa cells can be further damaged, thereby resulting in gastric cancer. Moreover, the CagA-positive genotype is significantly associated with an elevated risk of gastric cancer compared to the CagA-negative genotype (Ni et al., 2020). Hence, the International Agency for Research on Cancer has classified *H. pylori* as a class I carcinogen. Several studies demonstrate that the eradication of *H. pylori* reduced the incidence of gastric cancer (Choi et al., 2018; Kumar et al., 2020; Usui et al., 2023). According to a randomized intervention study, *H. pylori* treatment reduced risk of gastric cancer and had a cumulative protective effect on the mortality of gastric cancer after about eight years (Li et al., 2019). On the other hand, there are also studies showing that gastric cancer still developed after *H. pylori* eradication (Choi et al., 2014; Chen et al., 2016; Gong et al., 2023). Reasons for the development of gastric cancer may include significant endoscopic atrophy after eradication, histologic intestinal metaplasia, and long-term use of proton pump inhibitors (PPIs) (Shichijo and Hirata, 2018). Interestingly, Joo et al. (2019) believed that a dual role may be played by PPIs in the development of gastric cancer, and solid evidence must be obtained before observational clinical studies can be applied to establish PPI and its association with gastric cancer. Moreover, MALT lymphoma is another neoplastic illness caused by *H. pylori* infection. It spreads throughout the gastric mucosa because of uncontrolled proliferation of marginal zone B cells in lymphoid follicles (Salar, 2019).

### 2.2 Extragastric diseases

In extensive research, it was found that *H. pylori* infection not only causes gastric problems but also implicates dermatologic, hematologic, neurologic, and hepatobiliary system manifestations (Beydoun et al., 2018; Dardiotis et al., 2018; AlBalbeesi et al., 2021). An increasing body of evidence suggests *H. pylori* infection can increase the risk of colorectal cancer (Butt and Epplein, 2019; Zhuang et al., 2022; Guo et al., 2023). Based on analyses of large cohorts representing diverse populations, it was found that *H pylori* VacA-specific seropositivity is associated with an increased risk of colorectal cancer, particularly for African Americans (Butt et al., 2019). A more recent study has elucidated that *H. pylori* promotes colorectal cancer due to a reduction in regulatory T cells and pro-inflammatory T cells, as well as a signature of mucus-degrading microbiota (Ralser et al., 2023).

A number of skin conditions have been linked to *H. pylori* infection, including rosacea, chronic urticaria, lichen planus, atopic dermatitis, etc. Rosacea is a complicated skin disorder characterized by excessive inflammation and vascular dysfunction on the face. Much of the current literature has paid particular attention to the relationship between *H. pylori* infection and rosacea (Utaş et al., 1999; Szlachcic, 2002; Jørgensen et al., 2017). Data from a recent study revealed that 80.9% of patients with rosacea were infected with *H. pylori*. The infection rate was closely correlated with the severity of rosacea (Beridze et al., 2020). Its probable mechanism is that by generating cytotoxins and nitric oxide, *H. pylori* causes stomach mucosal inflammation and alters physiological processes, such as vasodilation, inflammation, and immunological modulation (Zhu et al., 2023). There is evidence that *H. pylori* contributes significantly to the pathogenesis of chronic urticaria (CU). Patients with CU (15/55 = 27%) were more prone to have *H. pylori* infection than those in the control group (6/55 = 10.1%), and *H. pylori*-positive patients had a six times greater odds of developing CU than in *H. pylori*-negative patients (Dennis et al., 2020). In addition, the eradication of *H. pylori* infection in patients with chronic spontaneous urticaria (CSU) can reduce gastrointestinal symptoms, improve the therapeutic response within two weeks, and reduce the chances of recurrence three months later (Guo et al., 2021).

Unexplained iron deficiency anemia (IDA) and vitamin B_12_ deficiency are also linked to *H. pylori* infection. Anemic patients with *H. pylori* infection in Pakistan were evaluated for the prevalence of IDA. There were 112 cases of anemia caused by *H. pylori* infection, including 42 cases of IDA (37.5%) (Rahat and Kamani, 2021). The results showed that adults who have IDA from previously unknown causes frequently have *H. pylori* infections. A meta-analysis also indicated that the treatment of *H. pylori* infections can improve anemia and iron status in patients with IDA who are infected with *H. pylori*, especially in those with moderate or severe anemia (Yuan et al., 2010). Vitamin B_12_ insufficiency is another hematologic condition connected to *H. pylori* infection. An *H. pylori*-infected patient is more likely to be deficient in vitamin B_12_ (Gökışık and Uyar, 2020). A study conducted by Ulasoglu et al. (2019) found that CagA-positivity and gastric inflammatory activity were significantly correlated with B_12_ deficiency.

Several lines of evidence suggest that persistent *H. pylori* infection can lead to neurologic disease, for example, Alzheimer’s disease (AD), Parkinson’s disease (PD), multiple sclerosis, and Guillain-Barré syndrome. AD is a neurodegenerative disease that causes progressive cognitive impairment. There was a close association observed between *H. pylori* seropositivity and AD mortality in men (Beydoun et al., 2018). Furthermore, another meta-analysis reported that patients with PD had a higher prevalence of *H. pylori* infection, which may deteriorate the clinical severity of the disease (Dardiotis et al., 2018). However, due to the ambiguous conclusions of these studies, the role of *H. pylori* infection in causing neurologic diseases remains unclear. For example, a previous study investigated the prevalence of *H. pylori* infection in Japanese patients with AD and found that there was no difference between them and the healthy control group (Shiota et al., 2011).

**Figure 1:**
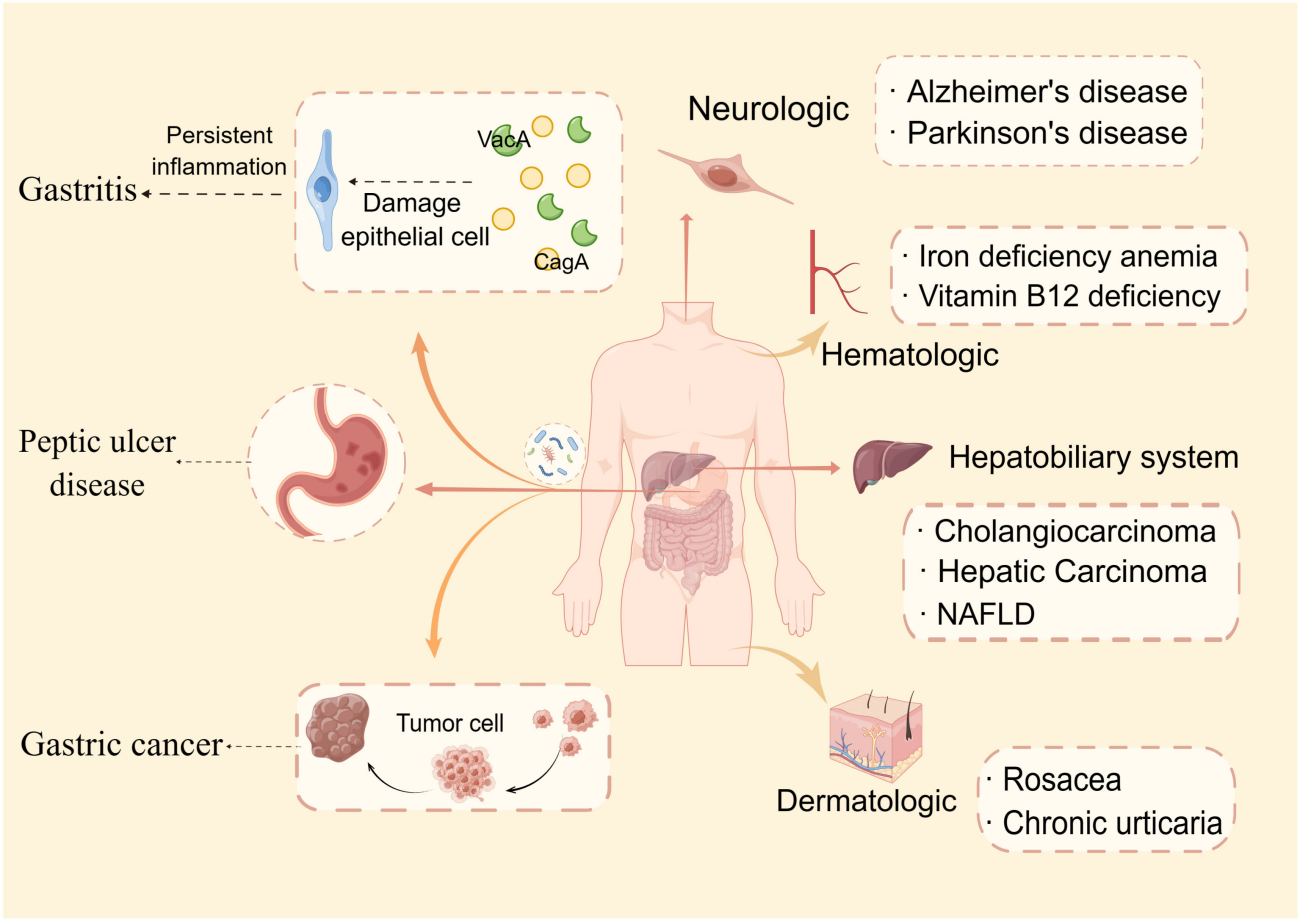
*Helicobacter pylori*-related diseases. This paper discusses two categories of *H. pylori*-related disorders: gastrointestinal (gastritis, peptic ulcer disease, and gastric cancer) and extragastric diseases. Extragastric disorders are further classified into four types: dermatologic, hematologic, neurologic, and hepatobiliary system manifestations.

Another extragastric disease associated with chronic *H. pylori* infection is hepatobiliary system disease. Previous reports demonstrated that the occurrence of cholangiocarcinoma is closely related to *H. pylori*. Dangtakot et al. (2021) found that long-term *H. pylori* infection and administration of *N*-nitrosodimethylamine can induce cholangiocarcinoma development in hamsters. CagA-positive *H. pylori* strains mainly contribute to the relapse and persistent advanced periductal fibrosis (Phung et al., 2022). In addition, Noto et al. (2022) demonstrated that increased *H*. *pylori*-induced injury within the context of iron deficiency is tightly linked to altered bile acid metabolism, with significant upregulation of deoxycholic acid, which may promote gastric carcinogenesis. Infection with *H. pylori* has also been related to non-alcoholic fatty liver disease, hepatic carcinoma, cirrhosis, and autoimmune liver diseases (Wang et al., 2015; Wang et al., 2022b; Yu et al., 2022). An *H. pylori* infection promotes liver inflammation, fibrosis, and necrosis and induces carcinogenic processes (Okushin et al., 2018). However, there is controversy regarding the relationship between *H. pylori* and liver diseases (Fan et al., 2018), and animal models are still needed to identify the specific mechanisms involved. In terms of treatment, aside from treating the underlying causes of hepatobiliary disorders, detection of *H. pylori* and anti-*H. pylori* agents should also be performed on patients with hepatobiliary diseases (**Figure 1**).

In addition to the pathogenicity of *H. pylori* in extragastric diseases, it also has a potential protective effect in some disorders, such as asthma. In a cross-sectional study that included 2241 participants, the rates of asthma diagnosis were 7.23% among *H. pylori*-negative children and 3.77% among *H. pylori*-positive children. This result indicates a significant inverse correlation between *H. pylori* infection and asthma (Wang et al., 2022a). This protective effect by *H. pylori* is supported by three hypotheses, including the gut-lung axis theory, the “disappearing microbiota” hypothesis, and the hygiene hypothesis (Strachan, 2000; Taube and Müller, 2012; Wypych et al., 2019). The mechanism involved may be related to the adjusting of Th1/Th2 and Thl7/Tregs balances, inhibiting dendritic cells, activating toll-like receptors, and reducing the expression of heat shock protein 70 (Zuo et al., 2021). Furthermore, asthma may be aggravated or incited by gastroesophageal reflux, with the potential mechanisms including an altered pressure gradient between the thorax and abdomen, parasympathetic reflex, and heightened bronchial reactivity (Solidoro et al., 2017).

## 3 Pathogenesis

### 3.1 Colonization

To successfully colonize the host and establish infection, *H. pylori* must be able to withstand an acidic stomach and adhere to host cells. Urea, a cytoplasmic enzyme, is largely responsible for *H. pylori*’s acid tolerance. Through catalysis, it adjusts the pH of the gastric environment by converting urea into carbon dioxide and ammonia. Nickel ions must be present in sufficient quantities in the stomach to ensure that one molecule of active urease functions fully. A low level of nickel results in a reduced level of *H. pylori* survival and colonization due to the lack of urease activation. It was demonstrated that the pro-inflammatory properties of urease promote the transformation of endothelial cells into reactive oxygen species (ROS)-dependent differentiation programs that contribute to *H. pylori* infection (de Jesus Souza et al., 2019). In addition to its strong association with various diseases, such as gastritis and peptic ulcer, previous research has established a link between the *H. pylori* urease and extragastric diseases, such as AD, which relates to the pro-inflammatory activity of urease and activation of the immune system, causing neuroinflammation and tau phosphorylation (Uberti et al., 2022). Using urease inhibitors to suppress urease function is, therefore, a potentially promising strategy for eliminating *H. pylori*.

Flagellar motility is an efficient way for *H. pylori* to enter the gastric mucus layer, and flagella can provide different motility modes to facilitate colonization depending on the medium in which the bacteria reside. The bacterial flagellar motor, which drives rotation of the flagellum for motility, plays a key role in bacterial survival in different environments (Tan et al., 2021). It drives flagellar rotation to achieve motion via two well-defined routes. The mode in which the flagellum lags behind the body when rotating counterclockwise is called the thruster mode and that in which the body lags behind the flagellum when rotating clockwise is called the traction mode (Antani et al., 2021). Aside from their motile functions, flagella also promote biofilm formation. Biofilms are adherent aggregates of microorganisms encased in a hydrated matrix of extracellular polymeric substances. They protect bacteria against antibiotics and harsh environments. It has conclusively been shown that *H. pylori* is capable of modifying its phenotype when grown in a biofilm, altering its metabolism, and reshaping flagella (typically locomotion organelles) into adhesive structures (Hathroubi et al., 2020). Moreover, prior studies have substantiated that the administration of active 2, an anti-motilin agent, effectively impedes the colonization of *H. pylori* in a murine model by suppressing flagellar motility. Consequently, this compound exhibits potential as an adjunct to the conventional antibiotic-based combination therapy for the treatment and eradication of *H. pylori* infection (Suerbaum et al., 2022).

**Figure 2:**
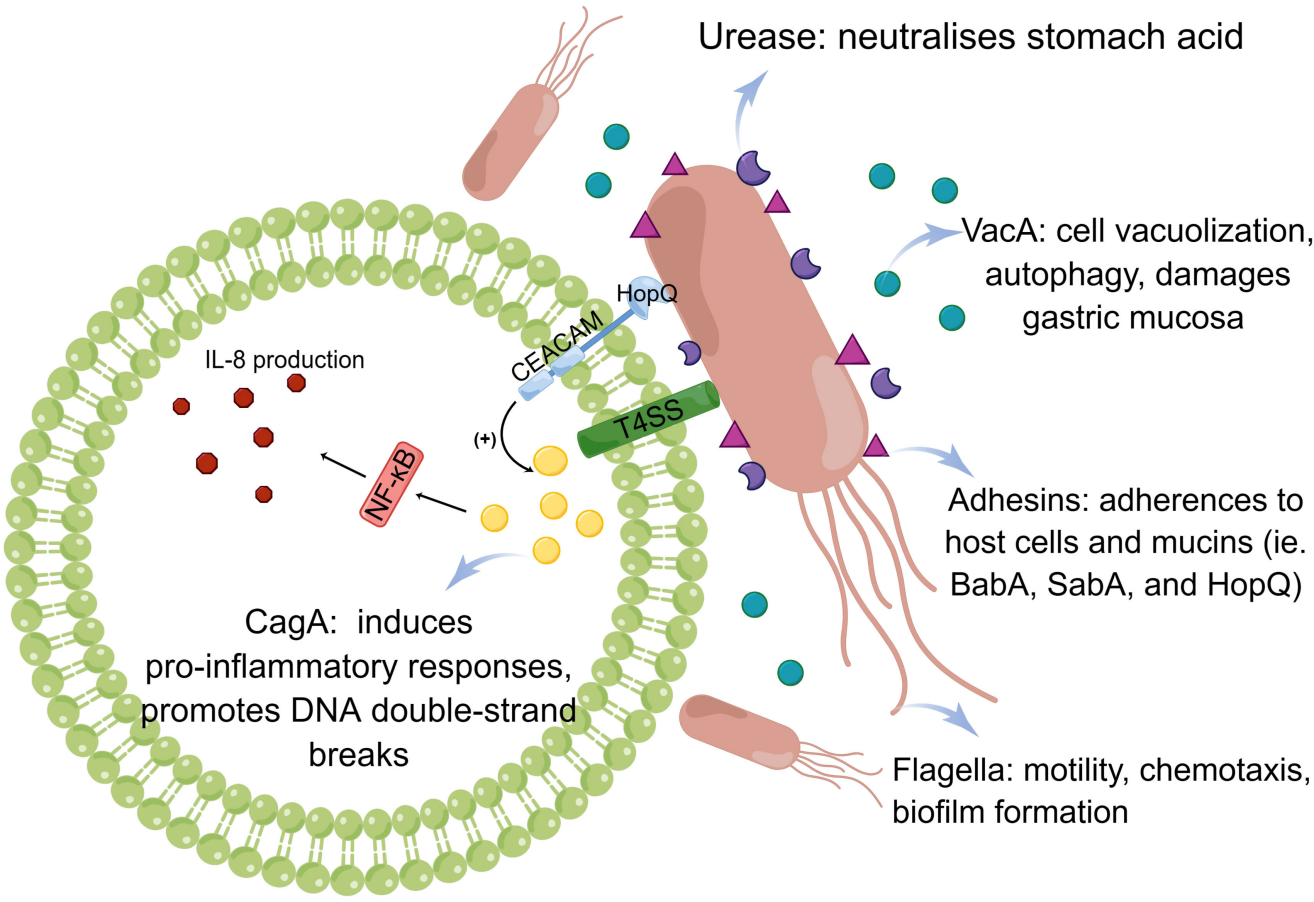
Pathogenesis of *Helicobacter pylori* infection. The pathogenic mechanism of *H. pylori* is based on its ability to colonize, which includes the action of urease, flagella, and adhesins. Virulence factors, such as CagA and VacA, play a distinct role.

Furthermore, adhesins plays important roles in *H. pylori* colonization. In the gastric mucosa, *H. pylori* adhesins (BabA, SabA, and HopQ) establish permanent colonization by binding to various mucins and receptors. BabA2 encodes a blood group antigen-binding adhesin (BabA) that allows *H. pylori* to bind to mucosal Lewis antigens, thereby facilitating colonization and determining bacterial density (Rad et al., 2002). HopQ is a conserved outer membrane adhesin that targets members of the carcinoembryonic antigen-related cell adhesion molecule (CEACAM) family, but overexpression of CEACAM molecules may contribute to cancer progression and recurrence. According to research by Moonens et al. (2018), the HopQ-CEACAM1 interaction may facilitate intracellular transport and phosphorylation of the CagA oncoprotein, which then induces the occurrence of disease. By studying adhesin modification of *H. pylori* using the general protein glycosylation system associated with lipopolysaccharide biosynthesis, it was found that the enzymes required for adhesin glycosylation are potentially better drug targets for the prevention and treatment of *H. pylori* infection (Teng et al., 2022) (**Figure 2**).

### 3.2 Virulence factors

*H. pylori* has a repertoire of virulence genes encoding effector proteins that can directly damage the gastric epithelium, such as CagA, VacA, duodenal ulcer promoting gene A protein, among others. Expression of a specific virulence factor can facilitate interactions between the host and the bacteria. It is important to note, however, that *H. pylori* virulence factors vary regionally, resulting in differing disease rates from country to country.

The cag pathogenicity island is a 40-kb chromosomal region containing the *CagA* gene and components of a type IV secretion system. This is only known to be present in *H. pylori* (Cover et al., 2020). CagA is located on the cag pathogenicity island and has been extensively studied. The bacterial type IV secretion system transports CagA into gastric epithelial cells, where it interacts with multiple host signaling proteins and undergoes tyrosine phosphorylation (Takahashi-Kanemitsu et al., 2020). Various *H. pylori* strains can be classified into two subtypes based on whether they are CagA-positive or -negative. They can dysregulate homeostatic signal transduction in gastric epithelial cells. For example, the CagA-SHP2 interaction leads to aberrant activation of SHP2, which deregulates Ras ERK signaling (Fujii et al., 2020). In addition to stimulating chronic inflammation of the gastric mucosa, CagA can also cause carcinogenesis through its ability to regulate apoptosis (Palrasu et al., 2022). Imai et al. (2021) have proposed that *H. pylori* CagA induces genomic instability through the promotion of DNA double-strand breaks (DSBs), potentially serving as a catalyst for bacterial gastric carcinogenesis. It is important to highlight that this finding contradicts that observed by Toller et al. (2011), where the cag pathogenicity island was considered unnecessary for DSB induction. However, it has been observed that CagA-positive strains exhibit greater motility and are linked to more severe clinical manifestations in patients compared with that in CagA-negative infections. For example, recent research has demonstrated that CagA-positive *H. pylori* strains, as opposed to CagA-negative ones, possess the ability to successfully colonize the gastric mucosa in mice and hinder endothelial function via the generation of ROS facilitated by CagA-containing exosomes (Xia et al., 2022).

Most *Helicobacter* strains contain VacA, a diffusible pore-forming exotoxin that induces vacuole formation in eukaryotic cells. The *H. pylori* academic community has provided a more precise and comprehensive description of the VacA genotypes. To date, various VacA genotypes have been identified, namely, s1, s2, i1, i2, i3, m1, m2, and m3 (El Khadir et al., 2020). In GES-1 cells, VacA can cause mitochondrial damage by altering the mitochondrial membrane potential, resulting in *PINK1*/Parkin-dependent mitophagy (Wang et al., 2022c). Additionally, it has been observed that VacA induces autophagy pathways in cells upon acute exposure. However, prolonged exposure of human gastric epithelial and mouse gastric cells to VacA leads to the promotion of defective autophagosomes and intracellular vacuole formation. These alterations facilitate the survival of *H. pylori* within host cells (Raju et al., 2012). Cytotoxic materials accumulate when autophagy is impaired or defective, resulting in DNA mutations, genome instability, and cancer development (Eslami et al., 2019). Furthermore, scholarly discourse has posited that VacA possesses the capability to selectively affect myeloid cells within the gastric mucosa, thereby triggering the upregulation of IL-10 and TGF-β in macrophages. This, in turn, hampers the initiation and efficacy of effector T-cell activation while fostering an environment conducive to the differentiation of regulatory T-cells in both the gastric mucosa and lungs of *H. pylori*-infected mice (Altobelli et al., 2019).

Virulence factors can interact and influence each other. VacA s1 strains, for example, also carry CagA, whereas almost all CagA-negative strains carry the less virulent genotype, VacA s2/m2 (Šterbenc et al., 2019). VacA as a co-stimulator for CagA phosphorylation can exert a synergistic impact and encourage CagA accumulation in gastric epithelial cells during *H. pylori* infection (Ansari and Yamaoka, 2020). Multiple virulence factors detected simultaneously increase the risk of gastric diseases. Therefore, future relevant studies should focus on eliminating the synergistic pathogenic effects of various virulence factors to eradicate *H. pylori*.

## 4 Diagnostic strategy

The accuracy of *H. pylori* detection is an essential part of the fight against infections caused by it and could greatly enhance public health. Based on whether endoscopy is needed, *H. pylori* detection methods can be divided into invasive and non-invasive examinations (**Table 1**).

**Table 1:** Comparison of different diagnostic strategies.

Diagnostic strategy		Advantage	Disadvantage
**Non-invasive examinations**	Urea breath test (UBT)	Easy to operate, low technical requirements for testers, high compliance	Patients taking proton pump inhibitors (PPIs) or H2RA may impact the accuracy of the results
	Stool antigen test (SAT)	Easily operated without oral administration of reagents and long-term sample collection cooperation	Storage, transport, and handling of fecal samples may influence the results
	Serological	No effect on gastric bleeding, PPIs, bismuth, and antibiotics	A positive serum test for antibodies cannot serve as the basis for ongoing infection
**Invasive examinations**	Endoscopy	Gastric pathology is evident	Accuracy and specificity of results vary considerably
	Histology	Higher sensitivity and specificity than those of the UBT and RUT	The abilities of observers have a significant impact on the analysis of results
	Culture	Extremely high specificity	Culture times are longer and the quality of culture conditions affects the results
	Rapid urease test (RUT)	Quick, easy, and accurate	Bacterial density and morphology in biopsies may affect the results
	Polymerase Chain Reaction (PCR)	Automated, simple, accurate, rapid, and efficient	Contaminated samples or samples containing PCR blockers affect the accuracy of the results

### 4.1 Non-invasive examinations

The urea breath test (UBT) is an effective method to detect *H. pylori* and is based on the ability of urease enzymes to convert a patient-ingested isotope-labeled urea solution into carbon dioxide and ammonia. Diagnostic accuracy of the UBT is high in people who do not have a history of gastrectomy and who have not recently taken antibiotics or PPIs (Best et al., 2018). As a result, many researchers recommend that PPI be ceased two weeks prior and antibiotics discontinued four weeks prior to UBT analysis (Malfertheiner et al., 2022). However, the results may return as false negative due to other factors. There is a significant reduction in UBT accuracy due to anti-acid drugs, such as lansoprazole, which accounts for 61% of ambiguous or false negative outcomes (Chey et al., 1997). Results by Capurso et al. (2006) support the hypothesis that the pattern of gastritis affects sensitivity of the UBT, and they have suggested that there is a high risk of false-negative results in patients with corpus-predominant gastritis. Additionally, as opposed to the high consumption of ^13^C, the ^14^C UBT is quick, simple, and relatively inexpensive. However, there are some limitations to this detection method, such as radiation exposure. In a word, the UBT is an excellent tool for assessing short-term *H. pylori* eradication after treatment because of its simplicity and high accuracy.

The stool antigen test (SAT) is also highly sensitive and specific, and applicable to all ages. For children, however, it is more effective to use monoclonal antibodies than polyclonal SAT (Zhou et al., 2014). There are two types of SATs, one based on enzyme immunoassay (EIA) and another on immunochromatography (ICA). The EIA-based tests are well-suited for assessing *H. pylori* infection in a substantial population. In contrast, ICA-based tests, due to their independence from specialized equipment, are convenient to conduct and suitable for economically disadvantaged regions. Additionally, it is worth noting that the EIA-based test exhibits a diminished level of precision and specificity compared to that of the ICA-based test (Shimoyama, 2013). Furthermore, the EIA-based test has a lower level of accuracy and specificity than that of the ICA-based test (McNicholl et al., 2020). The accuracy of SATs can also be affected by gastrointestinal bleeding and antibiotics, similar to that for UBTs. There is evidence that upper gastrointestinal bleeding reduces the diagnostic accuracy of *H. pylori* stool tests, especially their sensitivity (Peitz et al., 2003). Another disadvantage of the SAT is that it only detects current *H. pylori* infections, not previous ones.

A serological test can detect *H. pylori*-specific antibodies in serum, saliva, and urine. It is inexpensive, noninvasive, and convenient to detect IgG antibodies using laboratory-based serology, and is especially suitable for large-scale epidemic research. The presence of specific antibodies in the blood may be observed for several weeks after being infected with *H. pylori*. Hence, a positive serum test for antibodies cannot serve as the basis for an ongoing infection. In conclusion, serology is not recommended as a routine method for diagnosing *H. pylori* infection, but it can be helpful when combined with other methods.

### 4.2 Invasive examinations

The endoscopic examination is a common method used for detecting *H. pylori* infections. It can detect many abnormalities, such as duodenal ulcers, gastric ulcers, and gastric antral nodules. A gastric biopsy obtained under endoscopy can be used to confirm *H. pylori* infection by performing invasive tests. It is well known that the diagnosis of *H. pylori* can be made by evaluating diffuse redness, spotty redness, and mucosal swelling with conventional endoscopy, as well as swelling of the areae gastrica with indigo carmine contrast staining (Kato et al., 2013). Endoscopic techniques incorporate magnifying endoscopy, narrow-band imaging, confocal laser endomicroscopy, and Fuji intelligent chromoendoscopy. There are different diagnostic criteria, advantages, and disadvantages associated with different technologies. Researchers analyzed the data of 127 patients and indicated that white light and linked color imaging have a diagnostic accuracy for active gastritis of 79.5% and 86.6%, respectively. Linked color imaging is particularly useful in patients who have already had an infection with inactive gastritis in the past (Ono et al., 2020). It is, however, not recommended to use endoscopic techniques independently because their accuracy and specificity differ greatly.

Histology is the earliest method for detecting *H. pylori* infection; staining histological sections allow bacteria to be identified. In addition to hematoxylin and eosin (HE) staining, Giemsa, Warthine-Starry, and immunohistochemistry staining are also common staining methods used. Akeel et al. (2021) conducted various staining techniques on a cohort of 50 individuals diagnosed with chronic gastritis and exhibiting minimal or atypical *H. pylori* infection. Results revealed that immunohistochemical staining exhibits superior sensitivity and specificity compared with those of HE and Giemsa staining methods. Furthermore, Giemsa staining is commonly used because of its simplicity, even though its sensitivity strongly depends on the density of *H. pylori*. However, the ability and experience of doctors may affect histology results, as could certain requirements for the tissue to be taken, including the location of biopsy tissue, number of bacteria, etc.

Culturing involves isolating and cultivating *H. pylori* from biopsy tissue, which is highly sensitive. With the isolation of *H. pylori*, phenotypic and genotypic characterization can be performed to gain a deeper understanding of the pathogen (De Francesco et al., 2010). Furthermore, the cultivation of *H. pylori* plays a remarkable role in investigating antibiotic resistance. The Maastricht IV Consensus Report has proposed that *H. pylori* culture and antibiotic susceptibility testing should be recommended in areas showing a primary resistance to clarithromycin exceeding 20% or following failure of second-line treatment (Malfertheiner et al., 2012). In clinical practice, however, it is not suitable for routine detection of *H. pylori* because of difficulties in sample transportation and bacterial culture.

The Rapid Urease Test (RUT) is a simple and cheap method for diagnosing *H. pylori* infection. Antrum mucosa and gastric body biopsies can be done simultaneously to improve the performance of *H. pylori* detection. It is also possible to improve the response time of results for endoscopic biopsy by doubling the number of tissues examined (Laine et al., 1996). Nevertheless, when bacterial density of the biopsy tissue is not appropriate or under the influence of antibiotics, a false negative reaction is likely to occur (Wong et al., 2014). PPI has a transient negative effect on the viability, morphology, and urease test of *H. pylori*. Thus, it is not recommended to exclusively use the RUT to diagnose *H. pylori* if a patient has taken a PPI in the past (Yakoob et al., 2005). Despite having many influencing factors, because RUT has a high specificity, it remains the first test choice for diagnosing *H. pylori*.

The Polymerase Chain Reaction (PCR) can detect *H. pylori* DNA in saliva, stool, gastric juice, and other samples, as well as virulence factor genes. It is less affected by factors, such as preservation and transportation of biopsy tissues, than in other invasive tests. Almeida et al. (2015) classified CagA, CagE, VacA, IceA, and BabA2 genotypes through PCR to investigate the correlation of *H. pylori* genotypes with gastric histopathology in the central region of a South-European country. Real-time PCR with different PCR kits showed excellent sensitivity and specificity in detecting *H. pylori* in gastric biopsies and the mutations associated with macrolide resistance (Bénéjat et al., 2018). However, false-negative results are more likely to occur when specimens are contaminated during the process of collection, amplification, and documentation or the sample contains PCR blockers. In comparison with simple PCR, nested PCR is capable of amplifying DNA from samples that contain fewer target molecules, and accurate primer design and gene selection can enhance its specificity and sensitivity (Sulo and Šipková, 2021).

## 5 Treatment

### 5.1 Traditional experiential therapy

#### 5.1.1 Triple therapy

Standard triple therapy is one of the most common therapeutic methods used for *H. pylori* eradication, which consists of a PPI and two antibiotics. Drugs commonly prescribed as PPIs include omeprazole, lansoprazole, pantoprazole, rabeprazole, and esomeprazole. The commonly used antibiotics include amoxicillin, clarithromycin, levofloxacin, metronidazole, and others. Typically, the regimen of standard triple therapy should be continued for 10–14 days until *H. pylori* is completely eradicated. Increased antibiotic resistance has made it harder to eradicate *H. pylori*, especially because of clarithromycin resistance, and clarithromycin triple therapy may no longer be an appropriate first-line empiric therapy (Malfertheiner et al., 2022). It should be noted, however, that in areas with low clarithromycin resistance, this therapy is still prescribed as the first-line treatment. Standard triple therapy, consisting of clarithromycin, amoxicillin, and omeprazole for 14 consecutive days, can effectively eradicate *H. pylori* in rural areas of eastern Uganda (Lee et al., 2022). Studies documented that despite the unsatisfactory efficacy of standard triple therapy, vonoprazan triple therapy (vonoprazan, amoxicillin, and clarithromycin) achieves high eradication rates of over 90% and esomeprazole triple therapy (levofloxacin, esomeprazole, and clarithromycin) has the highest eradication rate in western countries (Rokkas et al., 2021). Vonoprazan is a novel potassium-competitive blocker with better acid suppression ability. However, further studies should be conducted to determine its efficacy in treating *H. pylori* on a global scale. In addition, a double-blind experiment in the USA indicated that rifampicin triple therapy (rifampicin, amoxicillin, and omeprazole) achieved an 83.8% eradication rate and had the potential for first-line empirical treatment of *H. pylori*. However, Asian patients were not included and the therapy caused partial adverse events (Graham et al., 2020).

#### 5.1.2 Bismuth quadruple therapy

In locations with a high prevalence of clarithromycin resistance (>20%), bismuth quadruple treatment is advised as the first-line therapy for *H. pylori* treatment (Malfertheiner et al., 2022). Usually, the PPI, bismuth, and two antibiotics make up bismuth quadruple treatment. Bismuth, a mucosal protective agent, exerts a bactericidal effect on *H. pylori* by inhibiting some of its enzymes, such as urease, fumarase, alcohol dehydrogenase, and phospholipase (Alkim et al., 2017). The short-term safety profile of bismuth is excellent and does not induce drug resistance effects. Pylera, a three-in-one capsule with bismuth potassium citrate, metronidazole, and tetracycline, has recently been accessible to streamline the administration process. Potassium bismuth citrate can be replaced with bismuth pectin (Cao et al., 2021). As the most commonly used antibiotic in bismuth quadruple therapy, tetracycline is becoming increasingly popular due to its efficacy and safety. Previous research has shown that a 10-day course of bismuth quadruple therapy achieved a 95% effective rate in the eradication of *H. pylori* in children, with a high compliance rate (Dutta and Phull, 2021). Bismuth-based quadruple therapy for 10–14 days also appears to be the most effective first-line option for penicillin-allergic patients. It is similar in efficacy and incidence of adverse events to the 14-day bismuth-containing regimen compared with that of the 10-day bismuth-containing regimen (Dutta and Phull, 2021). A few countries with *H. pylori* resistance, however, are unable to fully promote quadruple therapy for the treatment of *H. pylori* infections. Owing to the adverse reactions and treatment cycle associated with quadruple therapy, there exists a contentious debate among researchers regarding its popularity, necessitating further extensive and unbiased investigations. Moreover, the decision to employ bismuth quadruple therapy as the initial treatment for *H. pylori* should be contingent upon locoregional susceptibility testing, as well as the accessibility and cost-effectiveness of bismuth (Ouyang et al., 2022).

#### 5.1.3 Concomitant, sequential, and dual therapies

The three concomitant, sequential, and dual therapy regimens have names that correspond to how their respective medications are administered, such as sequential or combination administration. In research conducted in Myanmar, sequential therapy was just as efficient and well-tolerated as concomitant therapy (Myint et al., 2020). The simultaneous administration of four medications (a PPI and three antibiotics) during concomitant therapy, often referred to as “non-bismuth quadruple therapy,” can produce outcomes that are comparable with or superior to those of other forms. The Spanish Consensus meeting advocated for the first-line use of non-bismuth triple treatment (Gisbert et al., 2022). However, the complicated drug delivery methods employed by sequential and concomitant therapies may increase the prevalence of antimicrobial resistance globally. Concomitant therapy, in particular PPI, metronidazole, clarithromycin, and amoxicillin, is ineffective against dual clarithromycin-metronidazole resistance and ought to be discontinued as soon as susceptibility testing is widely used or when substitute treatments are made available (Graham et al., 2018).

PPIs and amoxicillin comprise dual treatment. In a location with high levels of clarithromycin resistance, a survey conducted in Japan revealed that the utilization of dual therapy, comprising vonoprazan at a dosage of 20 mg and amoxicillin at 750 mg twice daily, resulted in satisfactory rates of *H. pylori* eradication and comprehensive clinical efficacy that were comparable with those achieved through triple therapy involving vonoprazan. The dual therapy employed in this study involved the simultaneous administration of vonoprazan and amoxicillin (Suzuki et al., 2020). High-dose dual therapy, which raises a drug’s dose and administration frequency, has been developed to further enhance *H. pylori* eradication rates. According to a recent meta-analysis, high-dose dual therapy is the most effective treatment for *H. pylori* infection because it causes the fewest side effects (Zhu et al., 2020). Modified dual therapy with a high dose and frequent administration is just as effective, safer, and less expensive than quadruple therapy that contains bismuth (Yang et al., 2019). However, there are some discrepancies in these trials regarding the PPI used, dosage of the two medications, and the period between doses that still require more investigation. Going forward, it is critical to focus on preventing the emergence of resistance brought on by the high usage rate of amoxicillin.

### 5.2 Adjuvant therapy

#### 5.2.1 Probiotics

Efforts to eradicate *H. pylori* have steadily become more successful with the use of probiotics, which are living microorganisms. Probiotics, especially *Lactobacillus* and *Limosillactobacillus reuteri*, can combat *H. pylori* by secreting antimicrobial compounds, compete for binding sites, improve the function of the mucosal barrier, and boost the eradication effect (Ji and Yang, 2020). They can also control gut microbes to lessen negative effects and increase patient compliance. Bismuth and probiotics are an ideal combination for treating *H. pylori* infections. An earlier study displayed that 14-day high-dose PPI-bismuth triple probiotics treatment can be used as the first-line treatment regardless of the mechanism of *CYP2C19* and antibiotic resistance (Poonyam et al., 2019). However, the results of probiotic therapy vary widely, and clinical research is scarce in places such North America, Asia, Africa, and Europe. Future research on the best probiotic and antibiotic types and dosages to increase treatment efficacy of *H. pylori* infection should be theoretically supported by pertinent studies.

#### 5.2.2 Nanotechnology

Oral medicine is commonly used to treat *H. pylori*, but some antibiotics are degraded by the gastric environment, decreasing their effectiveness. For example, amoxicillin is degraded under the acidic pH of the gastric cavity (Hassan et al., 2019). Nanotechnology has been developed to protect and promote the targeted delivery of oral drugs. Nanomaterials have higher permeability and retention due to their small size. Moreover, it is possible to load more than two drugs at a time on a high surface area, allowing for collaborative treatment between more than two drugs (Rai et al., 2021). The identification of amoxicillin, when encapsulated in lipid nanoparticles, has demonstrated the potential to enhance drug retention at the infection site and shield it from adverse conditions within the stomach lumen (Lopes-de-Campos et al., 2019). Liposomes linolenic acid, a novel nanomedicine, can disrupt outer membrane barriers, lead to bacterial leakage, and deliver the drug to the proper location, which is a safe treatment method for *H. pylori* eradication (Zhang et al., 2020). Nevertheless, further examination should be done on the biodegradability and biocompatibility of the nanoparticles because there is very little information available about their toxicity and potential health hazards.

#### 5.2.3 Traditional Chinese medicine

**Figure 3:**
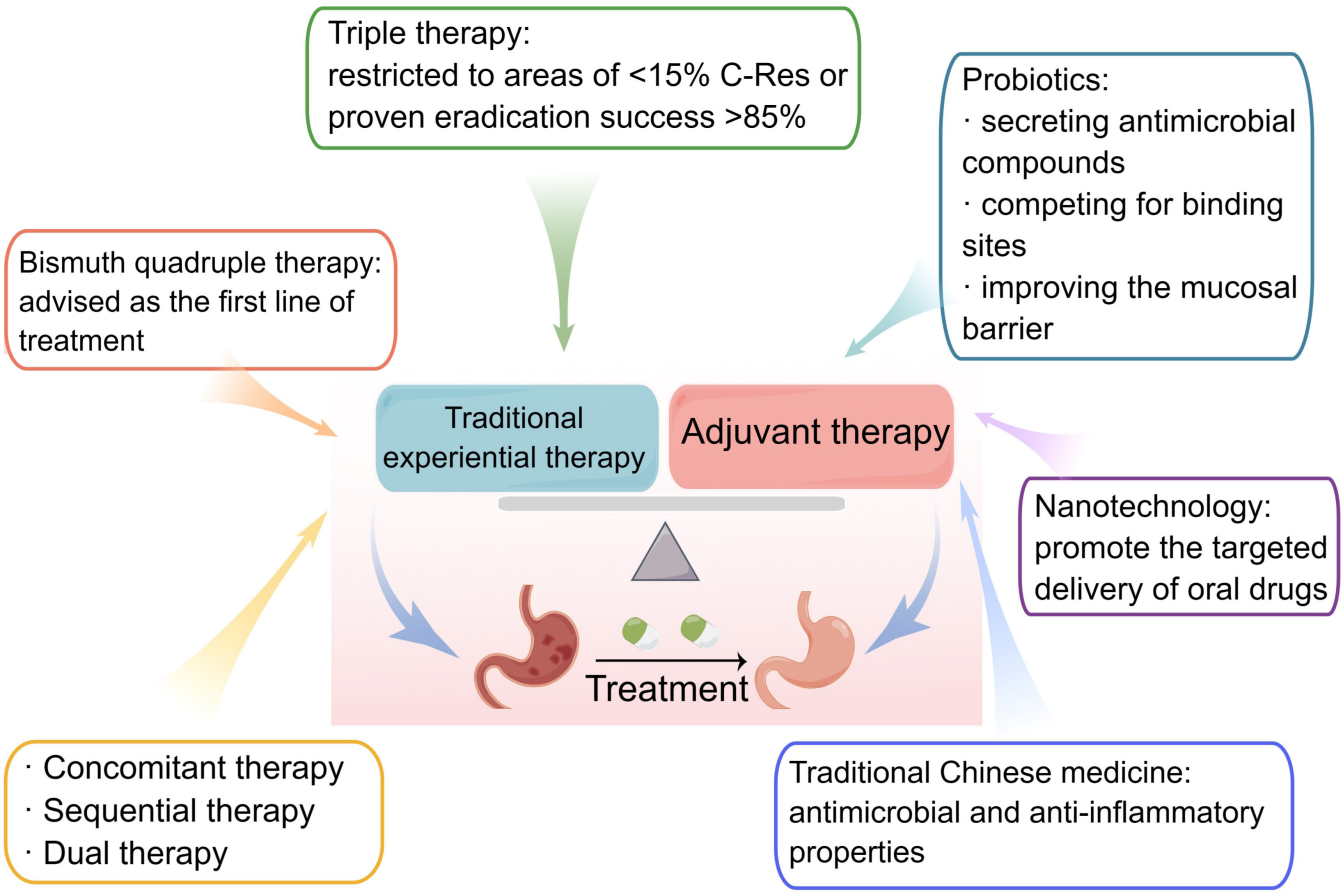
Treatment of *Helicobacter pylori* infections. Traditional experiential therapies for *H. pylori* include triple, bismuth quadruple, concomitant, and dual therapies. Probiotics, nanotechnology, and traditional Chinese medicine are all essential adjuvant therapies.

As TCM has been widely used in treating *H. pylori-*related gastrointestinal diseases, Chinese herbal formulae have apparently shown advantages in treating *H. pylori* infection. There is a large amount of clinical and pharmacological evidence showing that TCM has a curative effect on *H. pylori* and repeated infections (Guo et al., 2020; Liu and Lei, 2020). A study has shown that berberine, an active TCM ingredient, suppresses *H. pylori* infection, controls mucosal inflammation, and promotes ulcer healing (Liu et al., 2022). Additionally, it has been reported that monomeric compounds derived from TCM (coptisine, evodiamine, and patchouli alcohol) have antimicrobial and anti-inflammatory properties against *H. pylori*. The main mechanisms of action of TCM include suppressing the replication and transcription of *H. pylori*, reducing urease expression, destroying the bacterial structure, downregulating the expression of virulence factors, and inhibiting the activation of signaling proteins (Li et al., 2018; Xu et al., 2021; Yang et al., 2021). Several TCM prescriptions, including Shen-ling-bai-zhu-san and Huangqi Jianzhong decoction, have also shown the capability to treat *H. pylori* infections (Hu et al., 2022; Jin et al., 2022). Integration of the aforementioned TCM herbs and their active constituents with conventional experiential therapy has the potential to yield substantial augmentation in the eradication rate of *H. pylori* and the clinical efficacy in managing diverse gastrointestinal disorders associated with the bacterium. This outcome holds immense advantages for both patients in clinical settings and the overall global public health (**Figure 3**).

## 6 Drug resistance

With a limited selection of effective treatment options and extensive usage of certain antibiotics by the general public, rapid development of primary antibiotic resistance in *H. pylori* has become inevitable. From a clinical perspective, drug resistance leads to a significant reduction in the effectiveness of *H. pylori* treatment and an escalation in complications arising from *H. pylori* infection. Across all regions designated by the World Health Organization, primary and secondary resistance rates to clarithromycin, metronidazole, and levofloxacin exceed 15%, except for the Americas and South-East Asia regions in terms of primary clarithromycin resistance and the European region in relation to primary levofloxacin resistance (Savoldi et al., 2018).

**Figure 4:**
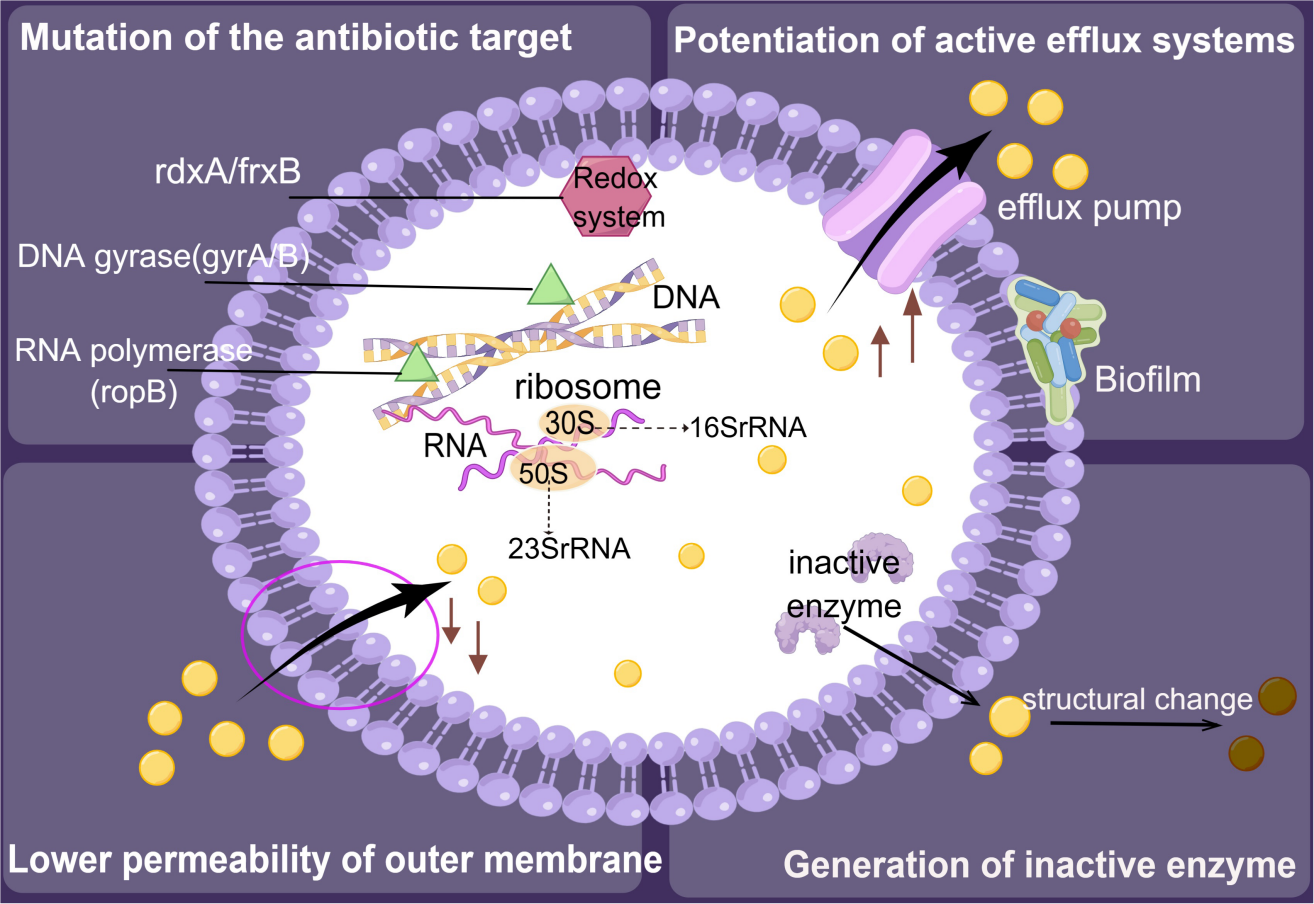
Resistance mechanisms of *Helicobacter pylori.* The development of resistance in *H. pylori* is primarily driven by mechanisms that involve the inactivation of enzymes, alteration of the target site for antimicrobial activity, reduction of outer membrane permeability, and enhancement of the active efflux system.

The primary mechanisms involved in acquiring bacterial resistance encompass the synthesis of inactivated enzymes, alteration of the antimicrobial target site, reduction of outer membrane permeability, and enhancement of the active efflux system (**Figure 4**). The formation of biofilms may enhance the survival of *H. pylori* and its resistance to antibiotics (Gong and Yuan, 2018). In addition, three types of drug resistance have been identified in *H. pylori*: single drug, multidrug, and heteroresistance (Tshibangu-Kabamba and Yamaoka, 2021). When it comes to single drug resistance, genetic structural alterations that impair the cellular function of antibiotics by either changing the drug target or by preventing drug activation within cells appear to be the primary cause of resistance in *H. pylori*. For instance, most metronidazole-resistant strains contain truncated *rdxA* and *frxA* genes that affect the synthesis of bacterial nucleic acids (Mannion et al., 2021). Furthermore, researchers found that a growing number of *H. pylori* strains have a multidrug resistance profile, such as simultaneous resistance to three or more drug families. Importantly, multidrug resistance has adverse effects on cancer treatment. Kopecka et al. (2020) suggested that lipids modulate multidrug efflux pump expression and activity, contributing to multidrug resistance in cancer. Heteroresistance occurs when bacterial subpopulations grow differently in response to an antibiotic within the same strain. Results showed that 36.2% and 68.1% of heteroresistant genotype samples were resistant to clarithromycin and levofloxacin, respectively. In most cases, heteroresistant genotypes tend to show a susceptible phenotype for clarithromycin and a resistant phenotype for levofloxacin (Wang et al., 2021). However, in clinical settings, there is no standard protocol for treating infections caused by heteroresistant *H. pylori*.

Precision therapy can be implemented by understanding the molecular mechanisms of *H. pylori* using methods such as whole gene sequencing. There have been proposals for *H. pylori*-targeted photodynamic therapy. It has a significant antibacterial effect on *H. pylori*, and this approach shows great potential as an alternative antibiotic treatment (Im et al., 2021). It is imperative for clinicians to possess a comprehensive understanding of the antimicrobial spectrum and indications for pharmaceutical agents, while exercising prudence in tailoring treatments to individual patients.

## 7 Other *Helicobacter* species

In the decades since its discovery in 1982, *H. pylori* has continued to broaden its range and enrich its species. The *Helicobacter* genus contains two types: gastric and enterohepatic *Helicobacter*. Human gastric infections with non-*H. pylori Helicobacter* species (NHPHs) have gained clinical significance in recent years. *Helicobacter* species other than *H. pylori* may colonize the stomach in humans and animals. Most of these agents in animals are transmitted to humans through direct or indirect contact with dogs, cats, and pigs (Taillieu et al., 2022). NHPHs can also be referred to as “*H. heilmannii*” and divided into “*H. heilmannii*” type 1 and type 2. The *H. suis* species is identical to “*H. heilmannii*” type 1, and “*H. heilmannii*” type 2 includes *H. felis*, *H. bizzozeronii*, *H. salomonis*, *H. cynogastricus*, and *H. ailurogastricus* (Ochoa and Collado, 2021).

In view of the difficulty of culturing NHPHs from the human stomach, diagnosis is frequently based on microscopy and molecular analysis of biopsy samples. It is also possible to identify different gastric NHPHs by using the whole-cell ELISA method (Matsui et al., 2023). Despite other gastric *Helicobacter* species in the stomach causing many clinical conditions, they have been identified with a much lower prevalence. All *Helicobacter* species in the human stomach can be treated with the regular antibiotic regimen, unless they exhibit antibiotic resistance.

## 8 Conclusions

This article provides a comprehensive overview of recent research on *H. pylori* infections, encompassing seven distinct areas of investigation. Despite significant advancements in our understanding of the disease since its initial identification, further exploration of its underlying mechanisms is imperative. The prevalence of *H. pylori* isolates varies considerably across different regions, potentially contributing to the severity of clinical manifestations. Consequently, it is crucial to ascertain the prevailing virulence factors in each specific locale to identify novel targets for therapeutic intervention. Moreover, a more accurate, less invasive, and simpler diagnostic method is required to clinically diagnose *H. pylori*. Of note, owing to the increasing resistance of *H. pylori* to antimicrobial therapy, it is necessary to focus on its resistance mechanisms and the development of vaccines, in addition to investigating new effective treatment options and drugs. Despite all of this, one potential issue with this review is that there are some points that have not been covered, especially in the diagnosis and treatment section. Only the essential components of each element that have been thoroughly investigated are selected for description, for example, the overview of virulence factors involved in the pathogenic mechanisms of *H. pylori*is far more extensive than those mentioned in the article. This paper does not comprehensively encompass certain *H. pylori* detection methods and treatment drugs, particularly those that are lesser-known and have not gained global recognition, such as natural remedies derived from specific ethnic communities.

## Author contributions

QS: Writing – original draft. CY: Writing – original draft. SZ: Writing – original draft. JL: Writing – original draft. MZ: Writing – original draft. XC: Writing – review & editing. HS: Writing – review & editing.
